# Radiotherapy-induced dynamic changes in the lymphocyte-to-monocyte ratio in patients with laryngeal cancer indicate poor prognosis

**DOI:** 10.3389/fonc.2023.1234953

**Published:** 2023-10-10

**Authors:** Natalia Cichowska-Cwalińska, Michał Bieńkowski, Marta Popęda, Magdalena Dróżka, Jacek Rutkowski, Jacek Jassem, Renata Zaucha

**Affiliations:** ^1^ Department of Oncology and Radiotherapy, Medical University of Gdańsk, Gdańsk, Poland; ^2^ Early Phase Clinical Trials Centre, Medical University of Gdańsk, Gdańsk, Poland; ^3^ Department of Pathomorphology, Medical University of Gdańsk, Gdańsk, Poland

**Keywords:** lymphocyte-to-monocyte ratio (LMR), neutrophil to lymphocyte ratio (NLR), platelet to lymphocyte ratio (PLR), programmed death 1 ligand 1 (PD-L1), laryngeal cancer (LC)

## Abstract

**Aim:**

We hypothesized that markers of inflammation correlate with response to radiotherapy in patients with non-metastatic laryngeal cancer (LC). Our aim was to assess peripheral and local markers of inflammation including lymphocyte to monocyte ratio (LMR), neutrophil to lymphocyte ratio (NLR), platelet to lymphocyte ratio (PLR), infiltrating CD8+ lymphocytes (TILsCD8), and programmed death 1 ligand (PD-L1) expression.

**Methods:**

We performed a retrospective single-center analysis of LC patients administered definitive (R-RT) or postoperative radiotherapy (PORT). The primary endpoint was overall survival (OS) in relation to peripheral and local inflammatory markers and their dynamic changes during RT.

**Results:**

Study group included 215 patients (R-RT, n=116; PORT, n=99). The baseline (t0) NLR and LMR were significantly correlated with OS in the R-RT group. In patients with high and low NLR at t0, the five-year OS was 33% and 56% (p=0.010) and in high and low LMR at t0, the five-year OS was 56% and 27% (p=0.003), respectively. The LMR increase during R-RT predicted better prognosis: the five-year OS in high and low LMR was 57% and 31% at t2 (after 2 weeks of RT) (p=0.015), 49% and 26% at t4 (p< 0.001), and 50% and 25% at t6 (p=0.013), respectively. Multivariable analysis shows that the worse performance status (p=0.003), the presence of nodal metastases (p=0.0001), and low baseline LMR (p=0.049) in the R-RT group, and the presence of nodal metastases (p=0.035) and completion treatment on time (p=0.042) in PORT group were associated with poor prognosis. The PD-L1 expression had no significant prognostic value in any of the examined patients.

**Conclusion:**

The baseline LMR and its dynamic changes during R-RT and baseline NLR are independent prognostic factors in patients with nonmetastatic LC. PD-L1 expression and number of TILsCD8 have no prognostic value in R-RT and PORT group.

## Introduction

Laryngeal cancer (LC) is the second most common cancer of the head and neck (HNC) region globally, after oral cavity and lip cancers with 184,500 new cases and around 100,000 deaths per year (1). In this group surgery, followed by postoperative radiotherapy (PORT) or definitive radio-chemotherapy (R-RT) and recently approved immunotherapy remain the gold standard treatments for patients with nonmetastatic advanced LC.

Unfortunately, despite the presence of early symptoms, the majority of LC cases present with locally advanced disease (2–4). The tumor stage and location, as well as the patient’s age, and performance status significantly impact the treatment outcomes. The five-year local failure rates (five-year LFR), regional failure rates (RFR), and distant failure rates (DFR) are 11%, 6%, and 19%, respectively (5, 6).

Cancer patients show local and systemic immune alterations (7, 8); consequently, peripheral blood cells, including neutrophils, lymphocytes, monocytes, and platelets, represent easy-to-evaluate immune system markers to impact prognosis (9–14). Variations in their numbers were shown in several cancer types, including mesothelioma, pancreatic cancer, renal cell carcinoma, and non–small cell lung cancer (9–15). Whether these variations represent a surrogate for the increased tumor burden or a tumor-associated immunological process is unclear (9). Chemokines and other inflammatory cytokines can be produced by both cancer and host immune cells, and can promote carcinogenesis as well as tumor progression. Lymphocytes and monocytes are involved in cancer proliferation, tumor cell invasion, and metastasis (16, 17), and increased numbers of monocytes correlate with the immune tolerance of cancer (10).

The clearest relationship between pretreatment lymphocyte to monocyte ratio (LMR), neutrophil to lymphocyte ratio (NLR), platelet to lymphocyte ratio (PLR), and survival has been shown in patients with metastatic disease (10, 14, 15, 18, 19). The dynamic changes in these parameters during or after radiotherapy may also have prognostic value (20, 21). These phenomena have been investigated in nasopharyngeal cancers and breast cancers treated with either definitive or adjuvant radiotherapy (19, 21), whereas their impact in LC is unclear. In this study, we evaluated prognostic baseline LMR, NLR, and PLR value, and radiation-induced LMR value changes in patients with non-metastatic LC. We also assessed the correlation of immune markers in primary tumors on OS.

## Materials and methods

### Patients and data collection

The study group comprised 215 consecutive patients with nonmetastatic LC confirmed by the multidisciplinary head and neck cancer tumor board who underwent R-RT or PORT at the Department of Clinical Oncology and Radiotherapy of the Medical University of Gdansk between 2012 and 2018. We retrospectively analyzed medical records for clinical characteristics, demographic data, treatment parameters, laboratory values, and survival. We calculated the baseline LMR, NLR, and PLR from blood counts (CBC) obtained within 15 days before treatment initiation. We also assessed the changes in LMR after 2 (t2), 4 (t4), and 6 (t6) weeks from RT initiation. We also performed a retrospective analysis of infiltrating CD8+ lymphocytes (TILsCD8) and programmed death 1 ligand (PD-L1) expression status in the archival biospecimens of the untreated primary tumor.

### Pathological assessment

Formalin-fixed paraffin-embedded tissue blocks were collected from tumor resections or diagnostic biopsies of primary tumor. Tissue microarrays (TMA), comprising three representative tissue cores (1.5 mm in diameter) for each patient, were prepared using the Manual Tissue Arrayer MTA-1 (Beecher Instruments, Inc., USA). Non-neoplastic tissues (tonsil and placenta) served as positive and negative staining controls, respectively. The TMA sections were first stained with hematoxylin and eosin to verify the invasive neoplastic content within each core. Next, consecutive sections were stained with IVD-grade antibodies, anti-PD-L1 (SP263) and anti-CD8 (SP57), using the automated BenchMark ULTRA IHC/ISH system (Roche Diagnostics, Switzerland). Pathological evaluation was performed by a board-certified pathologist (MB). The immunohistochemical analysis of the tissue arrays were unblinded. PD-L1 expression was assessed in tumor cells (TCs). Cells with complete membrane staining were considered positive, and the proportions of positive cells in each core (rounded to 10%) were determined. For statistical analysis, the patients were divided into three groups: consistently negative (all cores with no positive cells), heterogeneous, and consistently PD-L1 high (const-high, all cores with >30% positive cells). The numbers of infiltrating CD8+ lymphocytes within the tumor parenchyma (stromal lymphocytes were excluded) were determined for each core. Next, for each patient, the mean number of TILsCD8 per 1.76 mm^2^ (i.e., the area of a single core) of invasive tumor was calculated and recorded semi-quantitatively. Scores of <5, 6–50, 51–199, and ≥200 lymphocytes per core were rated as immunoscores (IMs) of 0, 1, 2, and 3, respectively. For statistical analyses, patients were divided into two groups: TILsCD8-negative (IM = 0) and TILsCD8-positive (IM ≥ 1).

### Treatment

All patients were treated in accordance with the departmental guidelines based on international recommendations and multidisciplinary decisions (22–25). Board-certified specialists in head and neck radiation oncology contoured the required target volumes and then prepared radiotherapy plans in accordance with International Commission on Radiation Units & Measurements (ICRU) report 83. All patients received photon radiotherapy using intensity modulated radiation therapy (IMRT) or volumetric modulated arc therapy (VMAT). The total dose was 66 Gy for R-RT and 54 Gy for PORT, with fractional doses in the range of 1.8 - 2.2 Gy. Medical physicists designed the treatment plans using the Eclipse system. Concomitant chemotherapy consisted of cisplatin (DDP) given at a dose of 100 mg/m^2^ i.v. at three-week intervals or 40 mg/m^2^ i.v. once a week. The overall survival time was defined as the time from the start of treatment to the date of death or to the date of last follow-up contact for patients still living.

### Statistical analysis

Data were analyzed and visualized using the R computing environment (4.1.2) (26). Receiver operating characteristic (ROC) curves were plotted for LMR, NLR, and PLR vs. death using the “pROC” package to select the optimal cut-off values for further dichotomization (27). The associations between LMR, NLR, PLR, TILsCD8, PD-L1 status and clinicopathological characteristics were assessed using the Mann-Whitney-Wilcoxon for continuous variables and the chi-square test for categorical variables. The associations with OS, the primary end point, were evaluated using univariable and multivariable Cox regression models, and Hazard Ratios (HRs) and corresponding 95% confidence intervals (CIs) were reported. All variables with a statistically significant univariate association were included in the multivariate model. Differences in OS between groups were assessed using the log-rank test and visualized with Kaplan-Meier curves using the “ggplot2” (28) and “survminer” packages (29). A p value < 0.05 without multiple testing adjustments was considered statistically significant.

## Results

The baseline characteristics of the 215 patients are presented in **Table 1**. The study group included more men than women (79% vs. 21%) and the median age was 62 years (range 36 to 93). A total of 215 patients received R-RT and PORT. All patients completed the intended radiotherapy. In ten patients, the total RT time was prolonged due to serious treatment-related complications (seven in R-RT and three in PORT). After a median follow-up of approximately 5 years (57.3 months), the median OS for the whole group was 6.6 years (79.7 months). The five-year OS for all patients was 70%.

**Table 1:** Baseline characteristics of patients with laryngeal cancer treated with R-RT and PORT.

Characteristic		R-RT n=116 (%)	PORT n=99 (%)	Fisher P-value
*Sex*	Female	23 (19.8)	23 (23.2)	0.618
	Male	93 (80.2)	76 (76.8)	
*Age*	< 60ys	51 (44.0)	49 (49.5)	0.493
	≥ 60ys	65 (56.0)	50 (50.5)	
*AJCC 8th edition* *T stage*	T1-2	56 (48.3)	30 (30.3)	0.012
	T3-4	60 (51.7)	67 (67.7)	
*AJCC 8th edition* *N stage*	N0	67 (57.8)	49 (49.5)	0.334
	N1-3	49 (42.2)	48 (48.5)	
*ECOG*	0	52 (44.8)	40 (40.4)	0.581
	1,2	64 (55.2)	59 (59.6)	
*Medical comorbidities*	No	61 (57.6)	64 (64.6)	0.096
	Yes	55 (47.4)	35 (35.4)	
*Chemotherapy*	No	60 (51.7)	45 (45.5)	0.612
	DDP q3w	27 (23.3)	21 (21.2)	
	DDP weekly	28 (24.1)	32 (32.3)	
	Other	1 (0.9)	1 (1.0)	
*Total cisplatin dose*	≥ 200mg	34 (61.8)	33 (62.3)	1.000
	< 200mg	21 (38.2)	20 (37.7)	
*Completion of scheduled treatment*	No	7 (6.0)	3 (3.0)	0.348
	Yes	109 (94.0)	96 (97.0)	
*Steroid therapy* *during RT*	No	90 (77.6)	81 (81.8)	0.500
	Yes	26 (22.4)	18 (18.2)	
*Antibiotic therapy during RT*	No	51 (44.0)	63 (63.6)	0.004
	Yes	65 (56.0)	36 (36.4)	

AJCC, American Joint Committee on Cancer; ECOG, Eastern Cooperative Oncology Group scale; DDP, Cisplatin; R-RT, definitive radiotherapy; PORT, postoperative radiotherapy.

In univariate analysis the baseline performance status (ECOG ≥ 1; p = 0.0038), anemia (Hg<12.5g%; p = 0.02), and presence of nodal metastases (N ≥ 1; p = 0.00048) were significantly negatively correlated with treatment outcomes in the R-RT group. The only poor prognostic factor with statistical significance in the PORT group was any lymph node involvement (N ≥ 1 vs. N0, p = 0.043) (univariate analysis).

The DDP regimen (100 mg/m^2^ q3w or 40 mg/m^2^ q1) had no impact on OS in both groups. Patients in the R-RT group who received DDP at a total dose equal to or greater than 200 mg/m^2^ (n=34) showed markedly longer median five-year OS than patients who received less than 200 mg/m^2^ (n=21) (p<0.001). In the PORT group, the median OS was not achieved.

Significant univariable risk factors for the primary endpoint (OS) were entered as covariates in multivariable Cox regression models (**Tables 2**, **3**). Clinical variables independently associated with OS in multivariable analysis in the R-RT group included the baseline performance status (ECOG ≥ 1; 0.003), the presence of nodal metastases (p=0.0001), and baseline LMR (p=0.049). The only variables that were independently associated with five-year OS in multivariable analysis in PORT group were the presence of nodal metastases (p=0.035) and completion of planned treatment on time (p=0.042).

**Table 2:** Baseline inflammation biomarkers in laryngeal cancer - clinical and pathological in the R-RT group (n= 116).

					Univariate analysis		Multivariate analysis	
		N	m5yrOS	m5yrOS (month)	HR (95%CI)	cox p - val	HR (95%CI)	cox p - val
***PD-L1***	negative	13	54%	NA	Ref			
	heterogenic	11	27%	30.4	2.24 (0.77 - 6.50)	0.139		
	constant - high	3	100%	NA	0.00	0.999		
***TILs CD8***	negative	3	67%	NA	Ref			
	positive	23	48%	57.2	2.13 (0.28 - 16.39)	0.469		
***NLR t0***	low	59	56%	NA	Ref			
	high	57	33%	30.4	1.9 (1.15 - 3.13)	0.012	1.62 (0.83 - 3.17)	0.154
***PLR t0***	low	49	45%	53.3	Ref			
	high	67	45%	47.0	1.06 (0.65 - 1.75)	0.807	0.64 (0.35 - 1.15)	0.135
***LMR t0***	low	44	27%	31.8	Ref			
	high	72	56%	NA	0.48 (0.29 - 0.78)	0.003	0.53 (0.28 - 1.00)	0.049

CI, confidence interval; HR, hazard ratio; LMR, lymphocyte to monocyte ratio; m5yOS, median five-year overall survival; NLR, neutrocyte to lymphocyte ratio; OS, overall survival; PD-L1, percentage of cells expressing ligand for programmed death 1; PLR, platelet to lymphocyte ratio; PORT, postoperative radiotherapy; R-RT, definitive radiotherapy; TILs CD8, infiltrating CD8+ lymphocytes; low, below cutoff by ROC; high, above cutoff by ROC.

**Table 3:** Baseline inflammation biomarkers in laryngeal cancer - clinical and pathological in the PORT group (n= 99).

					Univariate analysis		Multivariate analysis	
		N	m5yrOS	m5yrOS (months)	HR (95%CI)	cox p - val	HR (95%CI)	cox p - val
***PD-L1***	negative	34	62%	NA	Ref			
	heterogenic	14	64%	NA	1.02 (0.36 - 2.85)	0.976		
	constant - high	7	71%	NA	0.64 (0.14 - 2.82)	0.551		
***TILs CD8***	negative	8	63%	NA	Ref			
	positive	47	64%	NA	0.86 (0.25 - 2.95)	0.814		
***NLR t0***	low	65	69%	NA	Ref			
	high	34	59%	NA	1.51 (0.76 - 3.0)	0.234	1.50 (0.62 - 3.63)	0.370
***PLR t0***	low	41	61%	NA	Ref			
	high	58	69%	NA	0.75 (0.38 - 1.47)	0.404	0.51 (0.22 - 1.16)	0.108
***LMR t0***	low	30	57%	NA	Ref			
	high	68	69%	NA	0.66 (0.33 - 1.31)	0.232	0.59 (0.24 - 1.50)	0.269

CI, confidence interval; HR, hazard ratio; LMR, lymphocyte to monocyte ratio; m5yOS, median five-year overall survival; NLR, neutrocyte to lymphocyte ratio; OS, overall survival; PD-L1, percentage of cells expressing ligand for programmed death 1; PLR, platelet to lymphocyte ratio; PORT, postoperative radiotherapy; R-RT, radical radiotherapy; TILs CD8, infiltrating CD8+ lymphocytes; low, below cutoff by ROC; high, above cutoff by ROC.

### Baseline inflammation biomarkers

Prognostic impact of baseline peripheral and local inflammatory markers are shown in **Tables 2**, **3**. Time-dependent receiver performance characteristics (ROC) curves revealed pretreatment cut-off values for NLR, PLR, and LMR in the whole group of 2.8, 129.7, and 2.2, respectively. Most of assessed patients had high (above cutoff by ROC) LMR and PLR pretreatment values.

**Figure 1:**
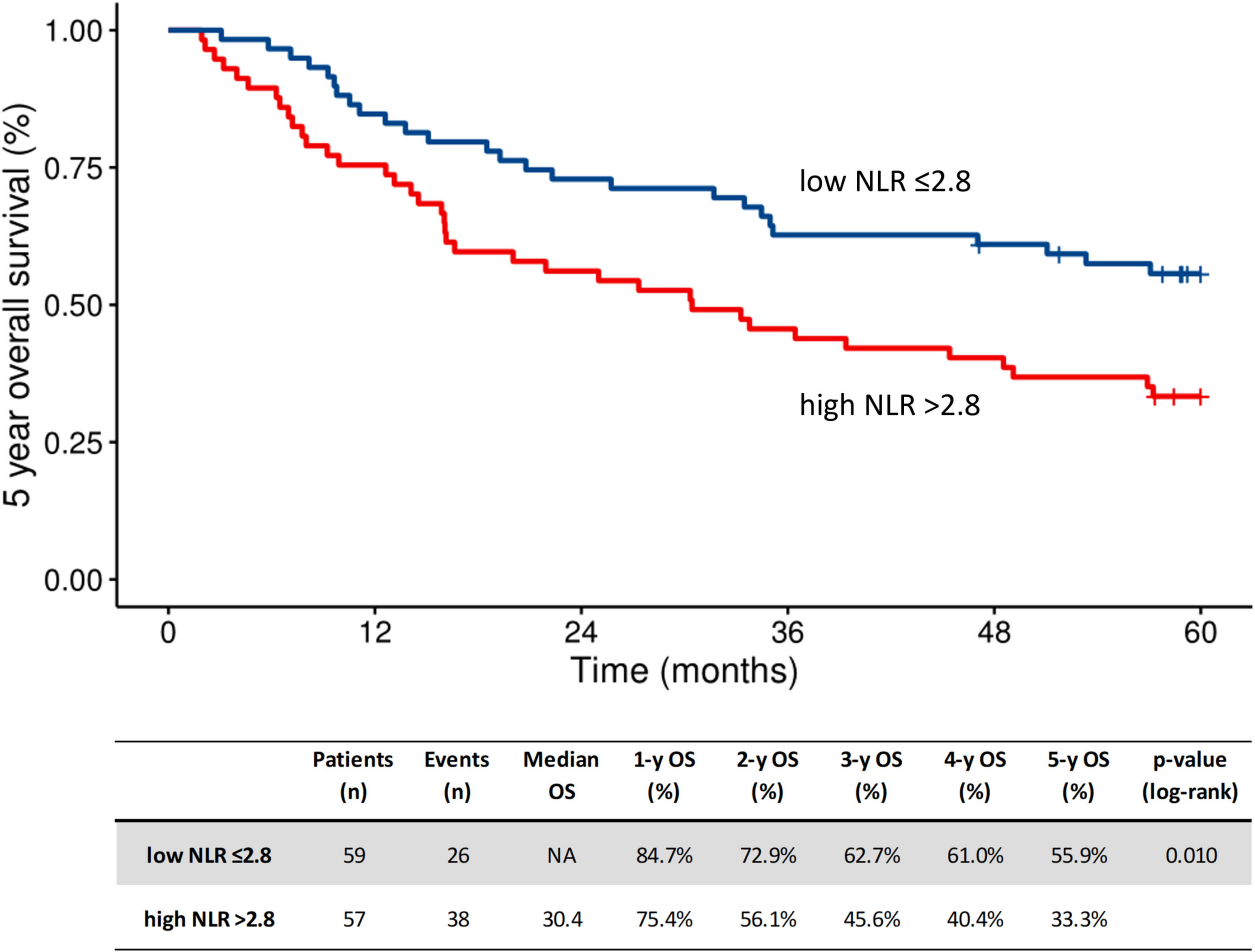
Kaplan-Meier plot of overall survival according to baseline NLR (t0) in the R-RT group. High, above cutoff by ROC; low, below cutoff by ROC; mOS, median overall survival; NLR, neutrocyte to lymphocyte ratio; R-RT, definitive radiotherapy.

The baseline high-NLR (t0) (n=57) value was correlated with worse long-term results in the R-RT group. The five-year OS in the high-NLR (n=57) and low-NLR (n=59) groups was 33% and 56%, respectively (HR 1.90; 95% CI 1.15–3.13 Cox p = 0.012) (**Figure 1**).

In the R-RT group, patients with high LMR (n=72) received higher cumulative doses of cisplatin than those with low LMR (n=44). The number of complications was 20% higher during RT, with more common antibiotic use in the low-LMR group than in the high-LMR group (p = 0.024). The PORT group did not show these differences. The PLR had no prognostic value in either group.

**Figure 2:**
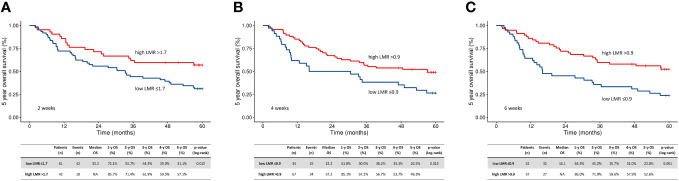
Kaplan-Meier plots of oncological outcome in relation to baseline LMR at two weeks **(A)**, four weeks **(B)**, and six weeks **(C)** after the start of radiotherapy - in the R-RT group. High, above cutoff by ROC; low, below cutoff by ROC; LMR, lymphocyte to monocyte ratio; mOS, median overall survival; R-RT, definitive radiotherapy.

In the R-RT and PORT groups, 51.6% (n=13) and 38.2% (n=21) of the patients, respectively, showed positive expression of PD-L1. The five-year OS was 80% for patients with const-high PD-L1 expression (HR=0.41, 95%CI: 0.09–1.75, p=0.218) vs. 60% for patients whose tumors were PD-L1 negative (**Figure 2**). The status of TILsCD8 positivity vs. negativity did not correlate with OS.

### RT-induced changes in LMR

**Figure 3:**
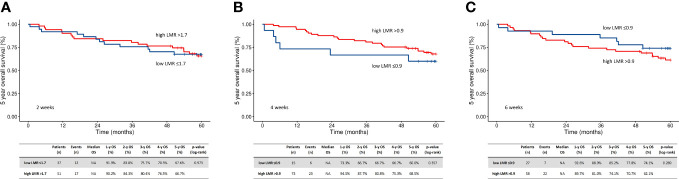
Kaplan-Meier plots of oncological outcome in relation to baseline LMR at two weeks **(A)**, four weeks **(B)**, and six weeks **(C)** after the start of radiotherapy - in the PORT group. High, above cutoff by ROC; low, below cutoff by ROC; LMR, lymphocyte to monocyte ratio; mOS, median overall survival; PORT, postoperative radiotherapy.

The ROC LMR cut-off points for all patients were 2.2, 1.7, 0.9, and 0.9 at the predefined time points (t0, t2, t4, and t6, respectively). At each time point, the patients were divided into four groups based on their t0 values (low vs. high LMR) and their RT-related changes (i.e., increasing or decreasing values). Decreases in LMR values were most common in the second week of treatment (**Table 4**). Low LMR during RT predicted worse OS, regardless of the baseline LMR value. The five-year OS rates for patients with low (n=61) vs. high LMR (n=42) were 31% vs. 57% at t2 (HR 0.51; 95% CI 0.29-0.89, p = 0.017), 26% (n=34) vs. 49% (n=67) at t4 (p < 0.001), and 25% (n=42) vs. 50% at (n=57) t6 (HR 0.53; 95% CI 0.31-0.88, p = 0.015), respectively. (**Figure 2**) Patients with a decrease in LMR at t2 had a median OS of 23.2 months compared to 57.2 months for those without an LMR decrease (p = 0.010). A further decrease in the low baseline LMR group was the worst prognostic factor (in the R-RT group). In PORT patients, the LMR and its changes had no prognostic value (**Figure 3**).

**Table 4:** RT induced LMR changes at time points, depending on the baseline LMR in the R-RT group.

		R-RT (n=116)			Univariate analysis	
		N	m5yrOS	m5yrOS	HR (95% CI)	Cox - p- val
			%	(months)		
***LMR 0***	**LMR 2**					
*high*	high	33	55	NA	Ref	
*high*	low	28	46	55.1	1.27 (0.62 - 2.61)	0.507
*low*	high	9	67	NA	0.70 (0.20 - 2.43)	0.577
*low*	low	33	18	27.3	2.42 (1.29 - 4.57)	0.006
***LMR 0***	**LMR 4**					
*high*	high	42	55	NA	Ref	
*high*	low	18	39	41.9	1.61 (0.77 - 3.39)	0.207
*low*	high	25	40	39.4	1.46 (0.74 - 2.87)	0.275
*low*	low	16	13	16.1	3.12 (1.56 - 6.26)	0.001
***LMR 0***	**LMR 6**					
*high*	high	35	57	NA	Ref	
*high*	low	23	35	30.4	1.87 (0.91 - 3.83)	0.086
*low*	high	22	45	52.9	1.29 (0.60 - 2.76)	0.508
*low*	low	19	11	15.9	3.79 (1.88 - 7.64)	0.001

CI, confidence interval; HR, hazard ratio; LMR, lymphocyte to monocyte ratio; R-RT, definitive radiotherapy, 0, time point before RT; 2, time point after 2 weeks of RT; 4, time point after 4 weeks of RT; 6, time point after 6 weeks of RT; low, below cutoff by ROC; high, above cutoff by ROC.

## Discussion

Since 1863, when Virchow first suggested a relationship between inflammation and cancer progression, malignant neoplasms have been seen as a mixture of interplaying cells, including cancer cells, stromal cells, infiltrating lymphocytes, dendritic cells, and even microbes (30). Any type of anticancer therapy will therefore have an impact on these cell types. RT is detrimental to cells capable of proliferation, making tumor cells and intra-tumoral immune cells vulnerable. We hypothesized that tumor cell apoptosis induces a local immune reaction, which further impacts the peripheral blood parameters responsible for the general immune reaction. Our assessment of NLR, PLR, and LMR in patients with LC confirmed that in non-metastatic LC managed with R-RT, the baseline and subsequent changes in LMR correlate with patient outcomes.

Immune peripheral markers have earlier been studied in different types of nonmetastatic and metastatic malignancies (31–35). Despite the use of different cutoff values (1.8 and 5.8) (31, 32), these studies have also shown a prognostic role for LMR. In our study, cutoffs for LMR were established at each of the predefined time points during treatment. Previous research on this topic in HNC focused on measuring this biomarker once before treatment. Further, patients with low baseline LMR were excluded (19, 35). We found that the decrease in LMR during RT had a significant prognostic value, regardless of the baseline LMR value. The RT-induced LMR changes were investigated in 2021 by Chia-Hsin Lin et al. in Asiatic patients with HNC, including a large number of patients with nasopharyngeal tumors (19). In that study, LC accounted for less than 8%. The dynamic LMR changes were calculated as the difference between LMR measured at baseline and during RT (called a delta-LMR). A low delta-LMR at the second week of RT was correlated with less favorable five-year OS (73% vs. 59%, p <0.001).

Studies on non-small cell lung cancer (NSCLC), colorectal cancer, and breast cancer have shown that higher LMR values after treatment initiation (compared to baseline values) correlated with better PFS and, sometimes with better OS (21, 36–38).

Few studies have investigated LMR changes in the PORT setting. Kim et al. showed a negative impact of low RT-related LMR on the PFS and OS at the second week of conventional RT (50.4Gy in 28 fractions) in breast cancer patients irradiated with a breast-conserving approach (21). Our study has not demonstrated similar results in the PORT group.

Our results confirm that several pretreatment factors, including performance status (ECOG ≥1), anemia, the presence of nodal metastases, and high NLR, significantly predict unfavorable outcomes in the R-RT group. The prognostic value of NLR has not been fully clarified. A large study including 5,700 patients with LC showed that high baseline value of NLR and PLR significantly correlated with OS. The NLR cut-off point was close to the value in our study (3.0) (39).

In our study patients with low or high baseline LMR values showed both an increase and a decrease in this parameter after RT. The worst prognostic results were obtained in the low-low LMR group and the best in the high-high group. Conversely, the OS in the low-high group was not worse than in the high-low group, placing both groups in the intermediate risk zone. Our results indicate that patients with a baseline low-LMR value, especially those with a decrease in LMR in the second week of radiotherapy had more complications and more frequently required antibacterial and antifungal treatment than the low-high group. These are possibly patients who require special attention (40).

We set the cut-off for PD-L1 expression at 50% of the tumor infiltration cells. The consistently positive expression in cancer cells (greater than 30% in all three cores for one case) clearly separated the survival curves compared to patients with higher point expression, but without statistical significance (p=0.073). We realize that our group is too small to draw far-reaching conclusions.

The prognostic value of PD-L1 expression has conflicting evidence in several cancers, including NSCLC, breast cancer, bladder cancer, and HNC (41–45). Previous studies of HNC have shown a correlation between PD-L1 overexpression with either poor prognosis (46, 47) or good prognosis (48, 49). Some clinicopathological features, e.g. tumor stage, or tumor site, and non-diabetic patients were correlated with positive PD-L1, but not with OS (50). There can be many reasons for this. First, the value of PD-L1 expression is dynamic in each patient, depending on the place of sampling, the time of its collection, patients comorbidities and possibly taken medications. Secondly, PD-L1 positive cells may negatively regulate the antitumor response of T lymphocytes, which has led to poor prognosis (51). On the contrary, TILsCD8+ infiltration may induce PD-L1 expression, which may be the reason for the association between high PD-L1 expression and good prognosis (52).

Clinical trials with the addition of immunotherapy, in various configurations, to definitive radiochemotherapy failed to demonstrate a statistically significant improvement (53–55). Nevertheless, anti-PD-1 immunotherapy plus chemoradiation is associated with a favorable trend toward improved PFS versus standard therapy in patients with locally advanced HNC. The benefit is noticeable in patients with PD-L1 combined positive score (CPS) >=1, especially with CPS >=20 (54). Additionally, there is a theory that radiotherapy and cisplatin has been shown to increase PD-L1 expression, and the concurrent inhibition of the PD-1/PD-L1 pathway may boost the antitumor activity of radiotherapy (56).

The pathomorphological guidelines of TILsCD8 assessment for HNC are partially derived from data on breast cancer (57). Assessing the infiltration of TILs, should take into account that the tumors arise from the squamous epithelium associated with lymphoid tissue. The preexisting background of lymphoid stroma makes TIL assessment in HNC challenging. Therefore, we focused on the evaluation of the intratumoral rather than stromal TILs. We used scoring of the IHC values to describe TILsCD8. We discarded cases diagnosed by biopsy from pathological lymph nodes to standardize our analysis. Unfortunately, we had archival pathological material from tumor tissues from only 84 patients. The status of TILsCD8 patients in our study did not have prognostic impact. A relatively small number of patients does not allow for firm conclusions. However, other studies showed that high levels of TILsCD8 correlated with improved outcomes in patients administered definitive chemoradiotherapy, whereas stomal TILs did not (58). In patients managed with PORT, a favorable correlation was also confirmed for both intratumoral and stromal TILsCD8 (59, 60). Nowadays, there are no uniform cutoff guidelines for the evaluation of PD-L1 expression and the technical aspects of TILsCD8 evaluation in LC patients.

The role of TILs in predicting the efficacy of chemoradiotherapy in LC may be clarified in large clinical trials. Establishing unambiguous predictive features of responses to immune therapies affecting the PD-L1/PD-1 pathway is also particularly important. These drugs are increasingly available for the treatment of patients initially inoperable, with relapsed or disseminated disease. In the future, this treatment is likely to be implemented in the adjuvant treatment.

This study possesses several inherent limitations. Firstly, its retrospective nature led to the omission of certain data points. The pool of suitable archival biospecimens was limited once we refined our selection to encompass only the highest quality materials. A subset of these biospecimens originated from patients who underwent treatment over 5 years ago. However, it is important to note, that all assessed samples have adhered to European Union standards for fixation and storage since 2010. We have duly considered and accommodated this aspect, making adjustments to our analysis and interpretations to mitigate the potential for erroneous negative outcomes. Lastly, it’s worth mentioning that we lacked access to data pertaining to disease-free survival or patterns of relapse.

## Conclusion

This study demonstrates that RT-inducted changes in LMR are associated with survival outcomes in LC patients administered definitive chemoradiotherapy.

## Data availability statement

The original contributions presented in the study are included in the article/supplementary material. Further inquiries can be directed to the corresponding author.

## Ethics statement

The studies involving humans were approved by Bioethical Committee of the Medical University of Gdańsk. The studies were conducted in accordance with the local legislation and institutional requirements. Written informed consent for participation was not required from the participants or the participants’ legal guardians/next of kin because this study has only the retrospective character.

## Author contributions

Guarantor of integrity of the entire study: NC-C. Study concepts and design: NC-C, MP, MB, and RZ. Literature research: NC-C. (Clinical studies) gathering the clinical data: NC-C and MD. (Experimental studies) data analysis: NC-C and JR. Statistical analysis: MP. Manuscript preparation: NC-C. Manuscript editing: NC-C, MB, MP, MD, JR, JJ, and RZ. First authorship: NC-C. Senior and last authorship: RZ. All authors contributed to the article and approved the submitted version.
